# Predictive factors for rapid progression in preterm neonates with necrotizing enterocolitis

**DOI:** 10.3389/fped.2022.970998

**Published:** 2023-01-09

**Authors:** Jiale Chen, Weitao Zhong, Longlong Hou, Tulian Lin, Junjian Lyv, Yan Tian, Zuyi Ma, Qiuming He, Wei Zhong

**Affiliations:** Department of Pediatric Surgery, Guangzhou Women and Children’s Medical Center, Guangzhou Medical University, Guangzhou, China

**Keywords:** preterm neonates, necrotizing enterocolitis, risk factors, rapid progression, predictive

## Abstract

**Background:**

Necrotizing enterocolitis (NEC) is a devastating gastrointestinal emergency with significant mortality and morbidity rates. A subset of patients progressed rapidly and underwent surgical intervention within a short period. This study aimed to establish a model to predict the rapid progression of NEC in preterm neonates.

**Methods:**

A retrospective study was conducted to review neonates with NEC between December 2015 and April 2019 at the Guangzhou Women and Children's Medical Center. Rapidly progressive NEC was defined as the need for surgical intervention or death within 48 h of NEC onset. Patients were divided into two groups: rapidly progressive NEC (RP-NEC) and non-rapidly progressive NEC (nRP-NEC). Data on demographics, perinatal characteristics, examination variables, and radiographic findings at onset were collected.

**Results:**

A total of 216 preterm neonates with NEC were included in the study, of which 64 had RP-NEC and 152 had nRP-NEC. The mortality rates of patients with RP-NEC and nRP-NEC were 32.8% and 3.28%, respectively. Male sex (*p*-value, adjusted odds ratio [95% confidence interval]: 0.002, 3.43 [1.57, 7.53]), portal venous gas (0.000, 8.82 [3.73, 20.89]), neutrophils <2.0 × 10^9^/L (0.005, 4.44 [1.59, 12.43]), pH <7.3 (7.2 ≤ pH < 7.3) (0.041, 2.95 [1.05, 8.31]), and pH <7.2 (0.000, 11.95 [2.97, 48.12]) at NEC onset were identified as independent risk factors for RP-NEC. An established model that included the four risk factors presented an area under the curve of 0.801 with 83% specificity and 66% sensitivity.

**Conclusion:**

Among preterm neonates with NEC, a significantly higher mortality rate was observed in those with rapid progression. It is recommended that close surveillance be performed in these patients, and we are confident that our established model can efficiently predict this rapid progression course.

## Introduction

Necrotizing enterocolitis (NEC) is a devastating gastrointestinal emergency that causes significant morbidity and mortality rates in neonatal intensive care units. Most patients with NEC can be treated conservatively, while 30%–50% must undergo surgical intervention. Patients who undergo surgery have a high mortality rate of 20%–50% (1). Of the non-surviving NEC patients, some died from a rapid course, called fulminant NEC, which was considered a sub-category condition. Fulminant NEC accounts for 6.7%–28% of the entire NEC population and is associated with a high mortality rate of 95.7%–100% (2–4).

The definition of fulminant NEC has been found to be inconsistent in previous studies. Fulminant NEC is defined as death due to NEC within 48 h following diagnosis (4), as NEC-totalis or death within 72 h of diagnosis (2), or as pan-intestinal necrosis or death within 48 h of onset (5). A rapid increase in feeding volume, increased concentration of milk fortifiers, lower birth weight, and earlier gestational age seem to be the risk factors for fulminant NEC. In addition, fulminant NEC is likely to lead to hematological abnormalities before disease onset or during disease episodes, such as a higher ratio of immature neutrophils, lower lymphocyte counts, and rapid development of thrombocytopenia and neutropenia (2, 4, 5).

Apart from the highly fatal sub-population NEC described above, attention should be paid to those with a rapid course and lower mortality. Lin et al. reported a sub-population of NEC, defined as patients who underwent intestinal surgical intervention within 48 h following NEC onset (6). Patients restricted to such a definition suffer from higher mortality than patients without a rapid course, but lower than that in a highly fatal sub-population, which is called fulminant NEC. To ensure effective and timely intervention in these patients, surveillance strategies should be more intensive than routine recommendations.

However, no model exists for predicting NEC with rapid progression at the time of NEC onset. Herein, we attempted to analyze the clinical characteristics of NEC with rapid progression and developed a model to efficiently predict rapid progression. The results obtained in this study may offer a novel predictive model for identifying neonates with rapidly progressive NEC and having the potential for clinical application. This new model is expected to help clinicians identify neonates with rapidly progressing NEC and timely surgical treatment to improve prognosis.

## Methods and materials

### Patients and data collection

This was a retrospective cohort study of all preterm neonates diagnosed with NEC Bell’s Stages II and III (7) at the Guangzhou Women and Children's Medical Center between December 2015 and April 2019. All patients were divided into a rapidly progressive NEC (RP-NEC) group and a non-rapidly progressive NEC (nRP-NEC) group. Rapidly progressive NEC was defined as patients who needed surgical intervention or who died within 48 h following NEC onset, while nRP-NEC was defined as patients treated only conservatively or those who underwent surgical intervention later than 48 h. Exclusion criteria were NEC after cardiac surgery, NEC after gastrointestinal surgery, or spontaneous intestinal perforation (SIP).

### Treatment of NEC

Medically, NEC was treated with bowel rest, gastrointestinal decompression, antibiotic administration, and nutritional support. Indications for surgical intervention include the following: (1) peritoneal free gas and severe infection; and (2) worsening of abdominal signs, infectious variables, and abdominal plain radiography findings during medical treatment.

### Statistical analysis

Data were presented as median [interquartile range] for abnormally distributed data, mean ± standard deviation for normally distributed data, and percentage for dichotomous variables. Univariate logistic regression analysis was performed to compare the variables between these two groups, and variables with *p*-values <0.2 were included in the multivariate logistic regression analysis to identify independent risk factors. The regression equation was expressed as ln (*P*/[1−*P*]) = constant + *β*1A + *β*2B + *β*3…, where *P* represents the predicted probability of rapidly progressive NEC. Beta 1, 2, and 3 represent the logistic regression coefficients of the independent risk factors (A, B, C, …), and receiver operating characteristic curve analyses were used to determine the diagnostic utility of the model. The results of the logistic regression analysis were presented as an odds ratio (OR) with a 95% confidence interval (CI). Statistical significance was set at *p* < 0.05. Receiver operating characteristic curve analysis was used to evaluate the optimal cutoffs, sensitivity, specificity, and predictive values. Statistical analyses were performed using SPSS 25.

## Results

**Figure 1:**
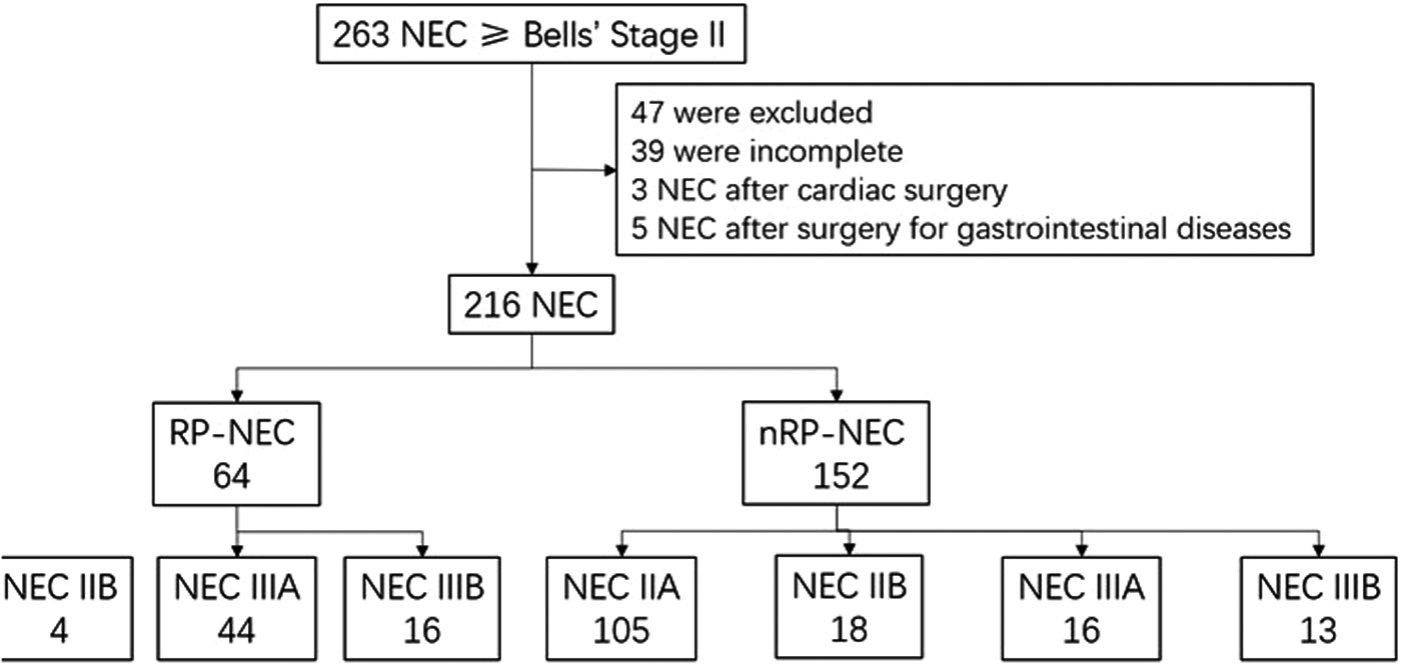
Flowchart for including patients.

A total of 263 patients diagnosed with necrotizing enterocolitis greater than or equal to Stage II were reviewed. Thirty-nine patients did not have accurate documentary records, three patients developed NEC after cardiac surgery, and five patients developed NEC after gastrointestinal surgery. A total of 64 and 152 patients were defined as having RP-NEC and nRP-NEC, respectively. In the RP-NEC group, four (29.63%) patients were diagnosed with Bell's Stage IIB, 44 with Stage IIIA, and 16 with Stage IIIB. Among patients with nRP-NEC, 105 were diagnosed with Stage IIA, 18 with Stage IIB, 16 with Stage IIIA, and 13 with Stage IIIB (Figure 1). The median time from NEC onset to surgical intervention was 24 h for RP-NEC (Table 1). The mortality rates in the RP-NEC and nRP-NEC groups were 32.8% (21/64) and 3.28% (5/152), respectively, with a mortality rate of 13.79% (4/29) for advanced NEC (IIIA and IIIB).

**Table 1:** Univariate and multivariate logistic regression analyses for determining the demographic features of RP- and nRP-NEC.

	RP-NEC (*n* = 64)	nRP-NEC (*n* = 152)	Univariate logistic regression		Multivariate logistic regression	
			*p*-value	Unadjusted OR 95% CI	*p*-value	Adjusted OR 95% CI
Male (*n* [%])	47 (73.4)	82 (53.9)	0.009	2.36 [1.25,4.48]	0.002	3.43 [1.57,7.53]
Gestational age (weeks)	31.79 (29.04,34)	33.22 (30.57,35.25)	0.050	0.91 [0.82,1.00]		
Birth weight (gram)	1612.84 ± 530.76	1772.65 ± 574.77	0.064	1.00 [0.99,1.00]		
Age at NEC onset (days)	10 (6,20)	8 (4,16)	0.053	1.02 [1.00,1.05]		
Time from onset to surgical indication (h) (median [IQR])	24 (12.33,40)	96 (73.5,130)				
Gestational diabetes mellitus (*n* [%])	18 (28.1)	27 (17.8)	0.089	1.81 [0.91,3.60]		
Gestational hypertension (*n* [%])	9 (14.1)	17 (11.2)	0.554	1.30 [0.55,3.09]		
Preeclampsia (*n* [%])	7 (10.9)	18 (11.8)	0.850	0.91 [0.36,2.31]		
Fetal growth restriction (*n* [%])	3 (4.5)	6 (3.9)	0.804	1.20 [0.29,4.94]		
Fetal distress (*n* [%])	5 (7.8)	10 (6.6)	0.745	1.20 [0.39,3.67]		
Premature rupture of membranes (*n* [%])	18 (28.1)	51 (33.6)	0.435	0.77 [0.41,1.47]		
Neonatal asphyxia (*n* [%])	9 (14.1)	12 (7.9)	0.168	1.91 [0.76,4.78]		
Small for gestational age (*n* [%])	5 (7.8)	18 (11.8)	0.384	0.63 [0.22,1.78]		
Cesarean delivery (*n* [%])	39 (60.9)	100 (65.8)	0.497	0.81 [0.44,1.48]		
Apgar scores (median [IQR])						
Apgar 1 min	9 (8,9)	9 (8,9)	0.339	0.92 [0.79,1.09]		
Apgar 5 min	9 (9,10)	9 (9,10)	0.742	0.96 [0.75,1.23]		
Apgar 10 min	9 (9,10)	9 (9,10)	0.866	0.98 [0.78,1.23]		
Hemodynamic patent ductus arteriosus (*n* [%])	7 (10.9)	10 (6.6)	0.282	1.74 [0.63,4.81]		

A trend of lower gestational age (median [interquartile range]; 31.79 (29.04, 34) vs. 33.22 (30.57, 35.25) weeks, *p* = 0.05), lower birth weight (mean ± SD; 1612.84 ± 530.76 vs. 1772.65 ± 574.77 g, *p* = 0.064), and late disease onset (median [inter-quartile range]; 10 (6, 20) vs. 8 (4, 16) days, *p* = 0.053) was seen in the RP-NEC group but were not statistically significant. This study demonstrated that male sex (*p*-value, unadjusted OR [95% CI]; 0.009, 2.36 [1.24, 4.48]) was positively correlated with RP-NEC in univariate logistic regression. In multivariable analysis, the odds ratio of male sex for RP-NEC increased by 243% (Table 1).

To analyze the correlations between postnatal administration and NEC course, we included details of feeding information, mechanical ventilation, blood transfusion, umbilical vein catheter, and administration of antibiotics, probiotics, caffeine, glutamine, and erythropoietin. None of the variables correlated with RP-NEC in either univariate or multivariate regression (Table 2).

**Table 2:** Univariate and multivariate logistic regression analyses for postnatal administrations of RP- and nRP-NEC.

	RP-NEC (*n* = 64)	nRP-NEC (*n* = 152)	Univariate logistic regression		Multivariate logistic regression	
			*p*-value	Unadjusted OR 95% CI	*p*-value	Adjusted OR 95% CI
Feeding information (*n* [%])						
Breast milk	7 (10.9)	19 (12.5)	1	reference		
Formula milk	36 (56.3)	54 (35.53)	0.464	1.70 [0.41,6.98]		
Combination	16 (25)	71 (46.7)	0.228	1.81 [0.69,4.74]		
No feeding	5 (7.81)	8 (5.26)	0.346	0.61 [0.22,1.70]		
Mechanical ventilation (*n* [%])	33 (51.56)	62 (40.79)	0.146	1.55 [0.86,2.78]		
Antibiotics (*n*/T [%])	29 (45.31)	58 (38.15)	0.328	1.34 [0.74,2.43]		
Red blood cell transfusion (*n* [%])	15 (23.44)	34 (22.37)	0.948	1.02 [0.51,2.04]		
Plasma transfusion (*n* [%])	4 (6.25)	18 (11.84)	0.222	0.50 [0.16,1.53]		
Caffeine (*n*/T [%])	24 (37.5)	61 (40.13)	0.718	0.89 [0.49,1.63]		
Probiotics (*n*/T [%])	5 (7.81)	12 (7.89)	0.984	0.99 [0.33,2.93]		
Umbilical artery catheter (*n*/T [%])	9 (14.06)	28 (18.4)	0.439	0.73 [0.32,1.64]		
Glutamine (*n*/T [%])	4 (6.25)	12 (7.89)	0.674	0.78 [0.24,2.51]		
Erythropoietin (*n*/T [%])	4 (6.25)	14 (9.21)	0.475	0.66 [0.21,2.01]		

RP-NEC, NEC with rapid progression; nRP-NEC, NEC without rapid progression.

In terms of radiographic findings, we demonstrated that portal venous gas was correlated with RP-NEC during univariable and multivariable regression analyses, with the odds ratio increasing by 781%. A white blood cell count <5*10^9^/L, hemoglobin <100 g/L, neutrophil count <2*10^9^/L, pH <7.3, and abnormal coagulation were positively correlated with RP-NEC in the univariable regression analysis. As presented in multivariable regression, neutrophils accounted for <2*10^9^/L, pH >7.2, but <7.3, and pH <7.2 increased the odds ratio for RP-NEC by 344%, 194%, and 1,095%, respectively (Table 3).

**Table 3:** Univariate and multivariate logistic regression analyses for radiographic findings and laboratory variables at disease onset of RP- and nRP-NEC.

	RP-NEC (*n* = 64)	nRP-NEC (*n* = 152)	Univariate logistic regression		Multivariate logistic regression	
			*p*-value	Unadjusted OR 95% CI	*p*-value	Adjusted OR 95% CI
Pneumatosis intestinalis (*n* [%])	41 (63.2)	85 (55.92)	0.269	1.41 [0.77,2.57]		
Portal venous gas (*n* [%])	25 (39.06)	13 (8.55)	0.000	6.85 [3.21,14.63]	0.000	8.82 [3.73,20.85]
White blood cell count						
5–20*10^9^/L	41 (64.1)	123 (80.92)	1	reference		
>20*10^9^/L	2 (3.13)	12 (7.89)	0.377	0.50 [0.11,2.33]		
<5*10^9^/L	21 (32.81)	17 (11.18)	0.000	3.71 [1.79,7.69]		
Hemoglobin <100 g/L	17 (26.56)	22 (14.47)	0.037	2.14 [1.05,4.37]		
Platelet count <100*10^9^/L	6 (9.38)	7 (4.61)	0.187	2.14 [0.69,6.65]		
Neutrophil count <2*10^9^/L	12 (18.75)	10 (6.58)	0.010	3.28 [1.34,8.04]	0.005	4.44 [1.59,12.43]
Abnormal coagulation (*n* [%])	32 (50)	51 (33.55)	0.024	1.98 [1.09,3.59]		
pH in arterial blood gas (*n* [%])						
≥7.3	42 (65.63)	137 (90.13)	1	reference	1	reference
≥7.2, <7.3	10 (15.63)	12 (7.89)	0.031	2.72 [1.09,6.74]	0.041	2.95 [1.05,8.31]
<7.2	12 (18.75)	3 (1.97)	0.000	13.05 [3.52,48.43]	0.000	11.95 [2.97,48.12]
Lactate >3 mmol/L (*n* [%])	25 (39.06)	54 (35.53)	0.622	0.86 [0.47,1.57]		
Procalcitonin <1.4 mg/L (*n* [%])	38 (59.38)	73 (40.03)	0.129	1.58 [0.88,2.86]		

To establish a model for predicting RP-NEC, male sex, portal venous gas, neutrophil count <2*10^9^/L, and pH <7.3 at NEC onset were included. The regression equation can be expressed as follows: ln (P/[1−P]) = 1.23 × Gender + 1.491 × Neutrophil count + 2.177 × portal venous gas [PVG]–2.481 × PH or 1.400 × pH–0.165. In this equation, P represents the predicted probability of RP-NEC, and sex is a dichotomous variable (0 = female; 1 = male). When a neonate with NEC had a portal venous gas and neutrophils count of <2*10^9^/L at NEC onset, the value of the neutrophil count and PVG was assigned to 1; otherwise, they were assigned a value of 0. In addition, when the PH of neonates with NEC was >7.2, but <7.3, the terms of the equation were 2.481 × pH, and when the pH was <7.2, the terms of the equation were 1.400 × pH; otherwise, the value of pH was assigned to 0 (Table 4).

**Table 4:** Multivariate logistic regression for determining the effects of gender, neutrophil count, portal venous gas, and pH in predicting RP-NEC.

Variable	*B*	SE	Wald	Sig	EXP (B)
Gender	1.233	0.401	9.469	0.002	3.43[1.57,7.53]
Neutrophil count	1.491	0.525	8.061	0.005	4.44 [1.59,12.43]
Portal venous gas	2.177	0.439	24.576	0.000	8.82 [3.73,20.85]
pH			14.986	1	reference
	−2.481	0.711	12.192	0.041	2.95 [1.05,8.31]
	−1.400	0.838	2.794	0.000	11.95 [2.97,48.12]
constant	−0.165	0.398	44.577	0.824	

The regression equation can be expressed as follows: ln (*P*/[1−*P*]) = 1.23 × Gender + 1.491 × Neutrophil count + 2.177 × portal venous gas [PVG]–2.481 × PH or 1.400 × pH–0.165. In this regression equation, P represents the predicted probability of RP-NEC, and sex is a dichotomous variable (0 = female; 1 = male). When a neonate with NEC has a portal venous gas and neutrophils count of <2*10^9^/L at NEC onset, the values of the neutrophil count and PVG are assigned to 1; otherwise, they are assigned a value of 0. In addition, when the PH of neonates with NEC is >7.2, but <7.3, the terms of the equation are 2.481 × pH, and when the pH is <7.2, the terms of the equation are 1.400 × pH; otherwise, the value of pH is assigned to 0.

**Figure 2:**
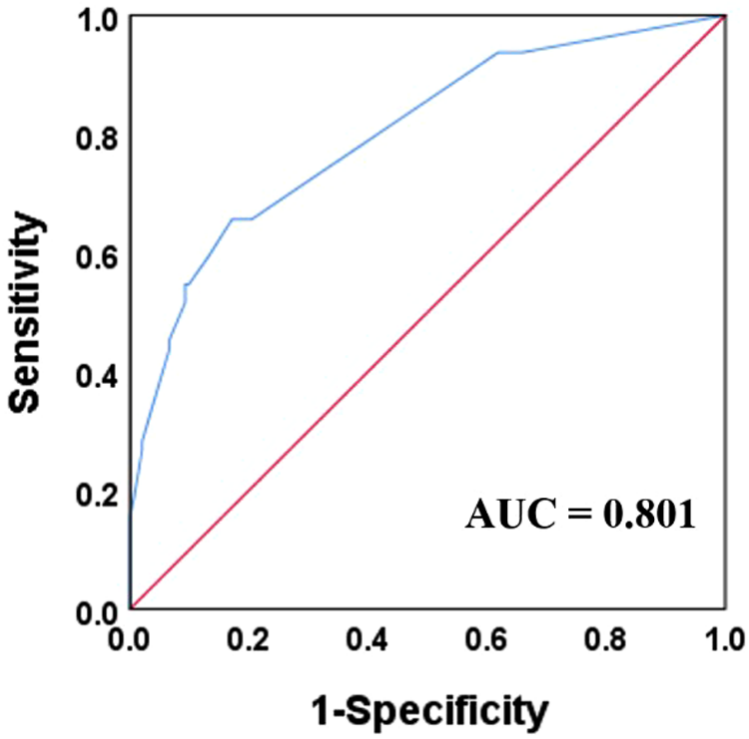
Receiver operation curve and area under curve (AUC) for the established model combined with male gender, portal venous gas, neutrophil count less than 2 × 10^9^/L, and pH value less than 7.3.

To evaluate the predictive value of this model, a receiver operating characteristic curve was constructed with an area under the curve (AUC) of 0.801, and the ideal cutoff value of *p* was 0.31, with 83% specificity and 66% sensitivity (Figure 2).

## Discussion

In this study, 29.6% of NEC patients with greater than or equal to Bell Stage II developed RP-NEC, and mortality was significantly higher in nRP-NEC. The incidence of RP-NEC was similar to that reported by Lin et al. (6). More importantly, the mortality rate in the RP-NEC group was higher (32.8%) than that in the nRP-NEC group (3.28%). Furthermore, male sex, portal venous gas, low neutrophil count, and low pH value were identified as independent risk factors, and all of them were included in a model that could present an AUC of 0.801.

In our study, gestational age and birth weight tended to correlate negatively with RP-NEC; however, they were not statistically significant in the univariate and multivariate regression analyses. In a retrospective cohort of 155 NEC, surgical NEC presented a significantly smaller gestational age and lower birth weight than medical NEC (8). Spontaneous intestinal perforation was also included in this cohort but was excluded from our study. Spontaneous intestinal perforation has been demonstrated to have a low gestational age and low birth weight, which might cause contamination (9). Getting rid of interference factors, such as SIP and suspected NEC, gestational age, and birth weight were reported as risk factors for severe conditions (3, 4, 10). We agree that they were related to final outcomes, but they were not promising factors to predict rapid disease progression within 48 h following the disease onset.

In this cohort, the age at NEC onset seemed to be higher in the RP-NEC group than in the nRP-NEC group, but this did not reach statistical significance (10 [6, 20] vs. 8 [4, 16], *p* = 0.053). In agreement with our study, Yu et al. presented a study showing that a severe condition had a tendency for a later age of onset (10 [7.5, 22.25] vs. 7 [2, 14], *p* = 0.4) (10). However, an earlier age of disease onset for severe NEC has been widely illustrated. El Manouni El Hassani et al. found that NEC onset days of surgical NEC occurred earlier than medical NEC, with a median of 10 vs. 15 days (*p* = 0.007), and a similar phenomenon was also illustrated in another study (10). The age-correlated severity of the disease may be explained by the fact that maturation of the intestinal barrier function is postnatal age-dependent (11). Patients with a lower postnatal age are vulnerable to pathogen invasion and disruption of immune homeostasis.

Gender has never received enough attention in NEC studies because most research has found that it is not a risk factor for NEC, although a few studies have reported a higher proportion of males among the NEC population (12, 13). However, there have been no reports that have focused on the effect of sex on the development of episodes of NEC. Herein, we found that male sex was correlated with RP-NEC, which worsened 48 h after clinical manifestation. The underlying mechanism of rapid progression in the male sex is not clear, but it is likely related to immunology disparity. The immune response in males seems more likely to cause tissue damage when attacked by pathogens, leading to a tendency to worsen. Umbilical vein blood from males releases more proinflammatory cytokines, interleukin-1β and interleukin-6, after stimulation with lipopolysaccharide, and males present an inadequate antioxidant defense than females (14, 15). Clinical research has also confirmed sex differences in neonatal mortality and morbidity outcomes (12, 16).

Portal venous gas, always accompanied by pneumatosis intestinalis, was found in 11.6%–33% of NEC patients (4, 17, 18). After disruption of the intestinal villus, luminal gas generated from bacterial fermentation broke into the intestinal wall, caused intravasation, and was then transported to the liver. Portal venous gas was considered to be a risk factor associated with worse prognosis, and approximately 48%–92% of PVG-positive NEC patients finally underwent urgent operation (3, 17, 18). Sharma et al. demonstrated that NEC with PVG had a higher operation rate, incidence of stricture, prolonged parenteral hyperalimentation, and cholestasis but not mortality (17). Chen et al. also found that the ratio of PVG detected by abdominal ultrasound or radiography was an independent risk factor associated with worse outcomes and was significantly higher in surgical or infants with NEC underwent surgical intervention or death (18). Lambert et al. demonstrated that PVG was more likely to be present in fulminant NEC, with 100% mortality (4). Although PVG per se is not an indication for surgical intervention, attention should be paid to this radiographic sign because our study showed that PVG-positive NEC patients were more likely to undergo surgery within 48 h. To date, no research has reported the time interval from identifying PVG to surgical indications. When there is a sign of PVG in a patient, we suggest a close evaluation of vital signs, abdominal signs, radiographic or ultrasonographic findings, and blood tests. No hesitate once there was any indication for surgical intervention.

The peripheral absolute neutrophil count (ANC) is a widely accepted index for predicting the severity of NEC. Patel et al. reported that 14 of 23 non-surviving NEC patients presented with neutropenia (ANC <1,500/μl) at the time of disease diagnosis, while 6 of 24 patients survived NEC (19). Hutter noted that non-surviving NEC presented a significantly lower ANC and 8 of 12 patients demonstrated neutropenia, while 2 of 28 patients survived NEC (20). Although the defined pathology of the decrease in ANC is not well illustrated, the migration of neutrophils to the inflammatory bowel or peritoneum is likely to be the predominant cause (21). Herein, we found that a lower ANC (<2 × 10^9^/L) was correlated with a rapid progression of NEC. A decrease in ANC indicated that neutrophils migrated to the inflammatory tissue or peritoneum with pathogen metabolism. Neutrophils are a common type of immune cell infiltration into lesions in acute inflammatory response, and immature neutrophils release numerous proinflammatory factors (22). Without mature immunomodulation, neonates are not efficient in constricting tissue damage, resulting in exaggerated inflammation and intestinal necrosis that require surgical intervention.

We also demonstrated that a lower pH value at disease onset was a predictor of RP-NEC. Our findings are comparable to those of previous reports. Linder et al. showed a close relationship between a lower pH value and NEC severity. In their retrospective cohort research concluded 20 infants with perforated NEC and 20 infants with non-perforated NEC, pH value on the day onset of NEC was significantly lower in infants with perforated NEC (7.27 ± 0.6 vs. 7.35 ± 0.5, *p* < 0.05) (23). Duci et al. and Yu et al. also found that the pH value was significantly lower in surgically perforated NEC (8, 10).

Because this was a retrospective study, bias or confounding factors could be possibly introduced. We did not analyze all known risk factors, including perinatal steroids, maternal cocaine, cigarette smoking, and maternal infection, because we did not record them regularly. In addition, some strategies such as the use of probiotics and milk fortifiers are not routinely used in clinical practice. Thus, we cannot take all of these into consideration.

## Conclusion

The mortality of RP-NEC progresses rapidly, and the need for surgical intervention 48 h after disease onset is significantly high. Thus, sufficient attention should be paid to the progress and onset of this disease, and appropriate treatment should be administered at an early stage to patients who are at risk for rapid progression at the time of disease onset. We found that a combination of factors such as male sex, portal venous gas, neutrophil count, and pH value was an effective model to predict RP-NEC with an AUC of 0.801. A retrospective case-control study identified risk factors that may indicate a rapid course in NEC patients. However, large, multicenter, prospective studies are required to determine the best perception in this subgroup of NEC.

## Data Availability

The original contributions presented in the study are included in the article/Supplementary Material; further inquiries can be directed to the corresponding author.
